# Circulating tumour DNA analyses reveal novel resistance mechanisms to CDK inhibition in metastatic breast cancer

**DOI:** 10.1093/annonc/mdy017

**Published:** 2018-01-17

**Authors:** C Abbosh, C Swanton, N J Birkbak

**Affiliations:** 1 Cancer Research UK Lung Cancer Centre of Excellence, London and Manchester; University College London Cancer Institute, London, UK; 2 Translational Cancer Therapeutics Laboratory, The Francis Crick Institute, London, UK

**Figure 1.**
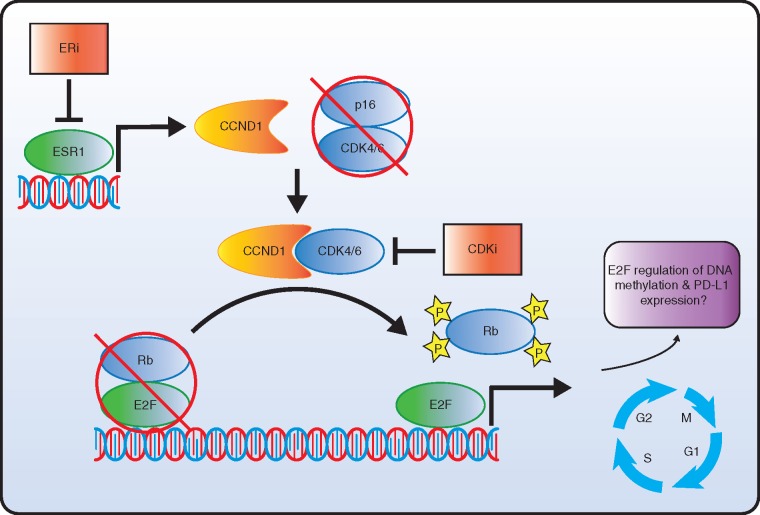
Cell cycle progression through E2F regulation, and the role of CDK and estrogen (ER) inhibitors. Transcriptional activation of cyclin-D1 (CCND1) through the estrogen receptor (ESR1), promotes dimerization of CCND1 and CDK4, and CCND1 and CDK6, escaping inhibition by p16. The cyclin-D/CDK complex phosphorylates Rb, releasing E2F to promote cell cycle progression through transcriptional activation of S-phase and G2/M gene sets. Additional transcriptional activation through E2F induction may affect genes involved in DNA methylation and PD-L1 expression. Pharmacological inhibition of ER and CDK4/6 synergistically affects downstream activation of E2F and inhibits cell cycle progression in the context of wild-type Rb. Mutational inactivation of Rb promotes therapeutic resistance.

Cyclin-dependent kinase (CDK) 4/6 inhibition has been demonstrated to improve progression-free survival (PFS) in patients with human epidermal growth factor receptor 2 (HER2−), hormone receptor positive (HR+) in advanced breast cancer [1–3]. Palbociclib, ribociclib and abemaciclib are orally bioavailable selective CDK 4/6 inhibitors. These small molecules likely bind the ATP-binding pocket within the CDK4/6 protein kinases thereby inhibiting phosphorylation of retinoblastoma tumour suppressor protein (Rb). In its hypophosphorylated state Rb remains bound to E2F thereby preventing progression through the G1-S-cell cycle checkpoint [4]. The mechanism behind the observed efficacy of CDK inhibition in metastatic breast cancer may relate to a dependence of HR+ breast cancer on CDK4/6 activity to override Rb mediated repression of cell cycle progression (Figure 1) [5].

CDK4/6 inhibitors have been approved by the US Food and Drug Administration (FDA) for initial endocrine therapy in postmenopausal women with metastatic or advanced HR+/HER2− breast cancer in combination with an aromatase inhibitor and for the treatment of endocrine therapy-resistant HR+/HER2− advanced or metastatic breast cancer in combination with Fulvesterant (a selective estrogen receptor degrader) [6]. In December 2017 the National Institute for Health and Care Excellence (NICE) has recommended CDK4/6 inhibitors in combination with aromatase inhibition as a first-line option for treating locally advanced or metastatic HR+/HER2− breast cancer [7]. Despite the success of the clinical studies that led to these recommendations, not all patients with HR+ breast cancer respond to CDK inhibition and a significant fraction progress within 2 years of initiation of treatment [1–3]. This underscores the need to identify mechanism of resistance to these targeted therapies to anticipate and target novel or subclonal resistance mechanisms driving breast cancer progression in these patients. 

Circulating tumour DNA (ctDNA) describes molecules of cell-free DNA circulating in plasma that originate from a patient’s tumour. ctDNA analyses by next-generation sequencing are demonstrating translational utility within clinical contexts ranging from non-invasive screening [8], tracking cancer burden and identifying residual disease in patients undergoing treatment of their disease [9–11] and identifying cancer associated mutations with therapeutic implications [12, 13]. In this edition of *Annals of Oncology* Condorelli et al. [14] leverage the ability of ctDNA analysis to interrogate the mutational landscape of progressive metastatic cancer to highlight loss of Rb function as a potential resistance mechanism to CDK4/6 inhibition. They provide a case-series of three patients treated at different institutions, by separate investigators, who developed progressive metastatic breast cancer following treatment with CDK4/6 inhibitors. In each case evidence of somatic alteration involving the *RB1* gene was noted through plasma ctDNA analyses at the point of disease progression. In the first patient a frameshift event involving exon 8 of *RB1* was observed that was predicted to result in a non-functioning truncated version of the protein. This event was not observed through NGS analysis of a liver biopsy acquired before CDK4/6 inhibition. In the second patient of the case-series four *RB1* alterations were noted at progression on palbociclib that were not detectable before initiation of therapy. The variant with the highest allele frequency in plasma at progression (Chr13(GRCh37): g.48937094G>A) has been previously shown in lung cancer to result in loss of the Rb protein region responsible for the binding of Rb to E2F-transcription factor complexes [15]. The final patient was observed to have a p.His483Tyr RB1 variant following ribociclib that is predicted to be deleterious.

This study is of interest for the following reasons. Firstly, it provides observational evidence of deleterious *RB1* alterations potentially being selected at disease progression following intervention with CDK4/6 inhibitors in patients with metastatic breast cancer. These observations build on a previous *invivo* investigation of CDK4/6 inhibitor resistance using patient-derived tumour xenograft models that suggested Rb1 inactivation as a resistance mechanism to chronic CDK4/6 inhibition [16]. Secondly, this study provides an early glimpse into the potential of ctDNA panels to detect acquisition of actionable alterations in patients who experience disease progression on anticancer therapy. Such a resource could inform mechanisms underlying resistance across a range of systemic therapies. There are advantages to ctDNA analyses as a research tool to understand the biology of heavily treated metastatic disease. The non-invasive nature of ctDNA examination overcomes barriers to tissue acquisition in late stage disease that include poor patient health, increased risk from biopsy procedures and cost.

There are however caveats to consider regarding this case-series. The number of patients described within the manuscript is small and there is no indication as to the frequency by which Rb1 alterations are detected at progression on CDK4/6 inhibition in this patient population. Additionally, patients 1 and 3 in the case-series were treated with two lines of therapy in between the biopsies showing lack of *RB1* alterations and ctDNA analyses demonstrating acquired *RB1* alterations—patient 1 received everolimus and exemstane before palbociclib and patient 2 received capecitabine and paclitaxel following ribociclib. Therefore, we cannot be certain that the acquisition of Rb1 alterations solely associate with selective pressure induced by CDK4/6 inhibition. Advancing the findings reported in this case-series will require a larger cohort to determine the incidence of Rb1 alterations as resistance mechanisms in patients with metastatic breast cancer on CDK4/6 inhibitors. Furthermore, more frequent ctDNA monitoring is necessary to follow the dynamics by which *RB1* alterations emerge and ascertain the association of their emergence with disease progression.

Given this work, it is notable that CDK4/6 inhibition has recently been associated with increasing tumour cell antigen presentation through a mechanism involving downregulation of Rb1-E2F induced DNA methyltransferase 1 (DNMT1) activity, increased expression of endogenous retroviral elements and type III interferon production [17]. This response to CDK4/6 inhibition was ameliorated by silencing of *RB1* and therefore could conceivably underlie an immune predatory selection pressure toward selection of Rb1 altered populations whilst undergoing treatment with CDK4/6 inhibitors. The fact that CDK4/6 inhibition has recently been shown to increase PD-L1 expression in mouse models of breast cancer provides a clear rationale for anti-PD1 treatment as a combination therapy with CDK4/6 inhibition before the emergence of Rb1 loss of function [18].
